# Case Report: Multiple extragenital adenomatoid tumors in the rectum and small intestinal mesentery

**DOI:** 10.3389/fonc.2025.1581770

**Published:** 2026-01-09

**Authors:** Zhongqian Wang, Lei Xiaoyan, Tianxu Fu, Zhenping Wang, Shishi Luo

**Affiliations:** 1 Department of Radiology, Hainan Affiliated Hospital of Hainan Medical University (Hainan General Hospital), Haikou, Hainan, China; 2 Department of Radiology, Hainan Hospital, Guangdong Provincial Hospital of Chinese Medicine, Haikou, Hainan, China

**Keywords:** adenomatoid tumor, extragenital, multiple, rectum, small intestinal mesentery, case report

## Abstract

Adenomatoid tumors (ATs) are benign tumors commonly found in the genital organs, usually occurring as a single mass with a good prognosis after surgery. However, multiple extragenital ATs are extremely rare, often asymptomatic, and may lead to misdiagnosis. This case report describes a patient with multiple ATs involving the rectum and small intestinal mesentery. The patient was a 39-year-old man who had been suffering from abdominal pain and recurrent loose stools for 6 months. Diagnostic contrast-enhanced computed tomography (DCE-CT) of the abdomen and pelvis showed multiple cystic–solid masses with uneven density and patchy enhancement at the edges. The patient underwent laparoscopic total excision, and postoperative pathology confirmed multiple extragenital ATs in the rectum and small intestinal mesentery. The patient recovered well, and no recurrence was observed during a 17-month follow-up. This case aims to raise awareness of the imaging features of multiple ATs in rare extragenital locations, preventing misdiagnosis, missed diagnosis, and excessive treatment.

## 1 Introduction

Adenomatoid tumor (AT) is a rare benign tumor originating from mesothelial cells. Because of its histological similarities with lymphatic or mesothelial cells, it was initially classified as “lymphangioma” or “mesothelioma” (1). In 1945, Golden and Ash first described its histological features and formally coined the term “adenomatoid tumor,” which is still used today (2). The formation of AT may be related to various factors, including genetic susceptibility, environmental influences, and other pathogenic factors affecting glandular tissues (such as chronic inflammation, diet, and smoking). These factors lead to abnormal proliferation of glandular epithelial cells through different pathogenic mechanisms. The exact pathogenesis of AT remains unclear, but studies suggest that its occurrence may be related to the regression of primitive mesothelium during the development of the gonadal Müllerian duct (3) or to molecular mechanisms. Goode et al. (4) found in a genetic analysis of 31 cases of reproductive system ATs that tumor necrosis factor receptor-associated factor 7 (TRAF7) was highly expressed, resulting in the phosphorylation of nuclear factor kB (NFkB), which, in turn, activates the NFkB pathway, causing an increase in L1CAM protein expression. Itami et al. (5) found that 37 out of 51 AT cases had TRAF7 gene mutations, and L1CAM protein expression was elevated. Another study (6) reported that 13 out of 18 ATs expressed L1CAM protein, while 24 malignant mesotheliomas were negative, suggesting that L1CAM protein expression can serve as a marker for AT and help differentiate it from malignant mesothelioma.

## 2 Case review

The study involving a human subject was reviewed and approved by the Medical Research Ethics Committee of Hainan General Hospital in accordance with the Helsinki Declaration. In this retrospective study, this patient’s written informed consent was obtained.

**Figure 1:**
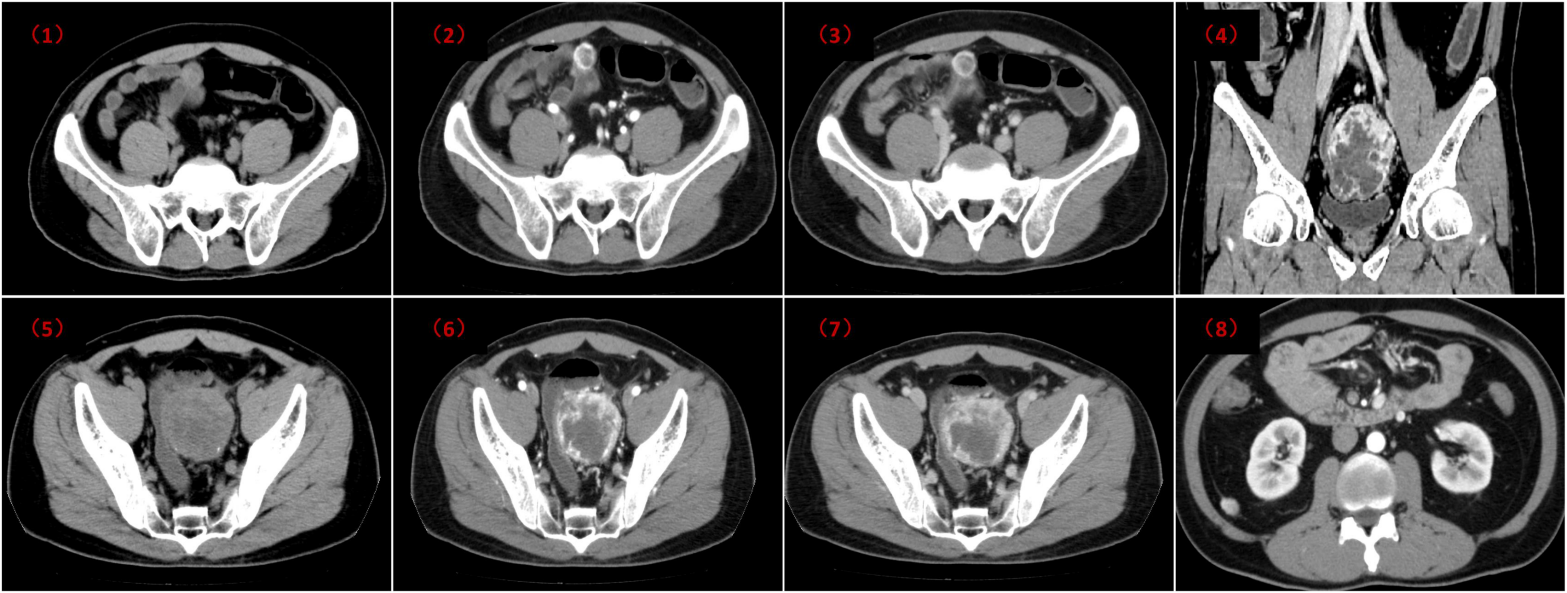
Contrast-enhanced CT of the abdomen and pelvis: (1) Non-contrast scan: A roundish, heterogeneous density cystic–solid mass is noted in the small bowel mesentery, with relatively well-defined borders (indicated by the red arrow). (2) Arterial phase: The periphery of the mass demonstrates significant enhancement, while the center shows mild, heterogeneous enhancement. (3) Venous phase: The periphery of the mass exhibits persistent marked enhancement, with washout of contrast in the central portion. (4) Coronal view: The anterior wall of the urinary bladder is slightly compressed (indicated by the green arrow). (5) Non-contrast scan: A heterogeneous density cystic–solid mass, measuring approximately 6.3 × 5.8 cm, is seen in the pelvic cavity adjacent to the rectum. Scattered punctate calcifications are noted at its margin, and the mass shows close proximity to the adjacent bowel loops (indicated by the purple arrow). (6) Arterial phase: The cystic wall/periphery of the mass shows significant enhancement, while the center shows no enhancement. (7) Venous phase: The cystic wall/periphery demonstrates persistent marked enhancement, with the center showing heterogeneous enhancement. (8) Enlarged lymph nodes are present in the abdominal cavity. The largest one measures approximately 1.5 cm in short-axis diameter and demonstrates moderate heterogeneous enhancement post-contrast (indicated by the yellow arrow).

The patient was a 39-year-old man who was admitted to the hospital on 1 July 2024, due to abdominal pain and recurrent loose stools for 6 months. Symptoms included narrowed or shapeless stools, increased bowel movements, lower abdominal pain before defecation, and relief after defecation, with no other abnormalities. Laboratory tests, including CEA, AFP, and CA19-9, were all within normal ranges. Diagnostic contrast-enhanced computed tomography (DCE-CT) of the abdomen and pelvis revealed multiple rounded cystic–solid masses in the right lower abdomen and pelvic cavity. The largest mass was located in the pelvic cavity, approximately 6.3×5.8 cm, with uneven density and patchy calcifications at the edges, in close relation to the adjacent intestine, and slight compression of the anterior wall of the bladder. The mesenteric mass had a CT value of 46HU on plain scan (**Figure 1**). During the arterial phase of the enhanced scan, the cystic wall of the mesenteric mass was significantly enhanced, while the center showed mild enhancement, with a CT value of 164/64HU [**Figure 1(2)**]. In the delayed phase, the cystic wall remained enhanced, while the central enhancement faded, with a CT value of 124/26HU [**Figure 1(3)**]. The rectal mass had a CT value of 33HU on plain scan [**Figure 1(5)**], with significant enhancement at the cystic wall during the arterial phase but no enhancement in the center, with a CT value of 169/22HU [**Figure 1(6)**]. In the delayed phase, the cystic wall showed persistent enhancement, and the center showed uneven enhancement, with a CT value of 118/110HU [**Figure 1(7)**]. The imaging characteristics of this case primarily demonstrated enhancement of the cystic wall within the mass, accompanied by thickening, exudation, and edema of the surrounding mesentery. Enlarged lymph nodes were observed in the abdominal cavity, with the largest measuring approximately 1.5 cm in short-axis diameter, showing moderate heterogeneous enhancement on contrast-enhanced scans. No ascites was present [**Figure 1(8)**]. The CT findings were suggestive of a neoplastic lesion, with mesothelioma considered a possibility.

**Figure 2:**
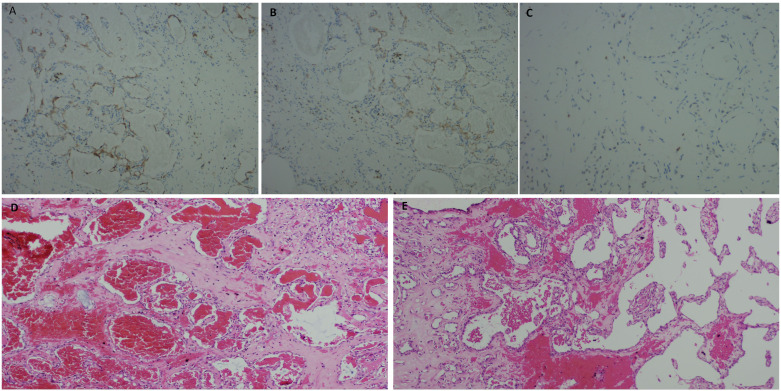
**(A)** Tumor cells are positive for CK5/6. **(B)** Tumor cells are positive for CR. **(C)** Tumor cells are positive for WT1. **(D)** The inner layer of the cyst wall is lined with epithelial-like cells arranged in a glandular structure, along with vascular proliferation of varying sizes. The lumen is dilated and contains mucus. **(E)** Areas of papillary structures are formed by glandular epithelium in the inner layer of the cyst wall. The vascular proliferation layer has a reticular pattern, and the stroma shows collagen fiber proliferation with infiltration of inflammatory cells.

On 3 July 2024, the patient underwent laparoscopic surgery under general anesthesia. Intraoperative exploration revealed exophytic masses on the rectum and the ileal mesentery. The masses were firm in consistency and adhered to the lateral abdominal wall with ill-defined borders. The rectal and small bowel mesenteric masses, along with the surrounding mesentery, were resected using an ultrasonic knife, and the involved intestinal segment was removed intact. The macroscopic appearance of the rectal mass showed a multilocular cyst, containing blood-tinged fluid, easy to peel from surrounding tissues. The small intestinal mesenteric mass showed irregular nodular features. Microscopic examination shows that the inner layer of the cyst wall of the mesenteric mass in the small intestine is lined with epithelial-like cells arranged in a glandular structure, along with vascular proliferation of varying sizes. The lumen is dilated and contains mucus (**Figure 2D**). In the rectal mass, areas of papillary structures are formed by glandular epithelium in the inner layer of the cyst wall. The vascular proliferation layer has a reticular pattern, and the stroma shows collagen fiber proliferation with infiltration of inflammatory cells (**Figure 2E**). Immunohistochemical staining results were positive for D2-40, CK5/6 (**Figure 2A**), CR (**Figure 2B**), WT1 (**Figure 2C**), and CD31, and negative for CD34 and CD117, with Ki67 approximately 1%. The final pathological diagnosis confirmed the rectal and small intestinal mesenteric masses as AT. The patient recovered well after surgery, with no recurrence or metastasis observed during a 17-month follow-up.

## 3 Discussion

AT is commonly found in the male and female genital organs such as the epididymis, fallopian tubes, and uterus. However, there are reports of atypical locations of single ATs. For example, Lao et al. (7) reported a 44-year-old woman who was admitted with intermittent abdominal pain and nausea/vomiting for over a month. DCE-CT showed a small intestinal tumor, and postoperative pathology confirmed an AT of the small intestine. Guan et al. (8) reported two cases of AT occurring in the adrenal glands, detected incidentally during physical exams, with postoperative pathology confirming AT. Goto et al. (9) reported a case of AT in the mediastinum in a 67-year-old woman, detected on a routine chest x-ray, and abdominal CT showed a thymoma. Postoperative histological and immunohistochemical examinations confirmed the diagnosis of thymic AT. These cases all involved single extragenital ATs, while multiple extragenital ATs in the rectum and small intestinal mesentery have not been previously reported. A PubMed search revealed only two cases of multiple extragenital ATs: one occurred in the liver and peritoneum (10), and the other occurred in the mesocolon and omentum (11). Both cases were asymptomatic and detected incidentally during imaging examinations, and postoperative pathology confirmed the diagnosis.

Imaging findings are crucial for diagnosis, as they closely correlate with histological features. The masses typically present as cystic, solid, or cystic–solid tumors and may be associated with secondary changes such as bleeding or calcification. On CT, the masses are usually round with clear borders. The cystic portions show low density, and when combined with hemorrhage or calcification, they may show high density. On enhanced scans, solid portions show enhancement, while cystic parts do not. On magnetic resonance imaging (MRI), solid tumors usually show uniform signal intensity, with slight variations on T1 and T2 scans. Cystic tumors show mixed signals, with the cystic portions showing long T1 and long T2 signals, while the solid portions show isointense T1 and slightly prolonged T2 signals. When combined with hemorrhage, both T1 and T2 signals are high, and on enhanced scans, solid portions show significant enhancement.

In summary, whether single or multiple, this disease is often difficult to differentiate from other conditions such as mesothelioma due to its atypical location and nonspecific imaging features. Therefore, such cases often require differential diagnosis with other abdominal masses. Common differential diagnoses include the following: (1) malignant mesothelioma: commonly associated with a history of asbestos exposure, presenting with ascites and abdominal pain, and showing irregular thickening of the peritoneum and uneven enhancement on CT or MRI; (2) cystic lymphangioma: typically occurs in the retroperitoneum, presenting as multi-locular cystic masses, with low density on CT and high T2 signal on MRI; (3) retroperitoneal hemangioma: usually large, with possible hemorrhage and necrosis, and uneven density/signal on CT and MRI, with typical vascular tumor enhancement on enhanced scans; (4) metastatic tumors: usually have a clear primary history, with unclear borders, uneven density, and signal, with common necrosis and infiltration on enhanced scans. In conclusion, although multiple ATs in the rectum and small intestinal mesentery are rare, imaging features such as uneven density, calcification at the edges, and non-uniform enhancement of the cystic wall can aid in preliminary diagnosis. After excluding conditions such as malignant mesothelioma, cystic lymphangioma, retroperitoneal hemangioma, and metastatic tumors, multiple AT should be considered.

## 4 Conclusion

Multiple extragenital ATs in the rectum and small intestinal mesentery are extremely rare. The clinical presentation is nonspecific, and surgical excision is the preferred and effective treatment method. The diagnosis depends on pathological examination. This case report enhances understanding of the imaging features and pathological characteristics of multiple ATs in these rare locations, helping clinicians with diagnosis and differential diagnosis, thereby achieving effective treatment.

## Data Availability

The datasets presented in this study can be found in online repositories. The names of the repository/repositories and accession number(s) can be found in the article/**Supplementary Material**.
