# Supplementary material for: Case Report: Multiple extragenital adenomatoid tumors in the rectum and small intestinal mesentery
Source: Front Oncol. 2026 Jan 9;15:1581770. doi: 10.3389/fonc.2025.1581770 (PMC12827149; doi:10.3389/fonc.2025.1581770)
Supplement: Supplementary file 1 [file DataSheet1.pdf]

CARE case report guidelines

# CARE Checklist of information to include when writing a case report

(cc) BY-NC-ND

| Topic | Item | Checklist item description | Reported on Line |
|---|---|---|---|
| Title | 1 | The diagnosis or intervention of primary focus followed by the words "case report" | adenomatoid tumors |
| Key Words | 2 | 2 to 5 key words that identify diagnoses or interventions in this case report, including "case report" | adenomatoid tumor, extragenital, multiple, rectum, small intestinal mesentery,case report |
| Abstract (no references) | 3a | Introduction: What is unique about this case and what does it add to the scientific literature? | The occurrence of this case in an atypical location and the fact that it is multiple are unique |
| | 3b | Main symptoms and/or important clinical findings | The main symptoms were abdominal pain and recurrent unformed stools. |
| | 3c | The main diagnoses, therapeutic interventions, and outcomes | An DCE-CT scan of the main abdominal basin revealed mesothelioma, which was surgically resected and pathologically confirmed as multiple adenomatoid tumors |
| | 3d | Conclusion—What is the main "take-away" lesson(s) from this case? | Adenomatoid tumors may also occur outside the genitals |
| Introduction | 4 | One or two paragraphs summarizing why this case is unique (**may include references**) | YES |
| Patient Information | 5a | De-identified patient specific information. | √ |
| | 5b | Primary concerns and symptoms of the patient. | Increased frequency of defecation, lower abdominal pain before defecation, ease after defecation |
| | 5c | Medical, family, and psycho-social history including relevant genetic information | The patient had no history of other cancers, no mental illness, and no similar family history |
| | 5d | Relevant past interventions with outcomes | The patient was found by chance during physical examination |
| Clinical Findings | 6 | Describe significant physical examination (PE) and important clinical findings. | The digital rectal examination was negative, and there was no tenderness, rebound pain, or muscle tension |
| Timeline | 7 | Historical and current information from this episode of care organized as a timeline | The patient had no history of treatment |
| Diagnostic Assessment | 8a | Diagnostic testing (such as PE, laboratory testing, imaging, surveys). | Abdominal pelvic enhanced CT indicates mesothelioma |
| | 8b | Diagnostic challenges (such as access to testing, financial, or cultural) | The patient's disease site is not typical, and the diagnosis of multiple diagnoses is difficult |
| | 8c | Diagnosis (including other diagnoses considered) | mesothelioma |
| | 8d | Prognosis (such as staging in oncology) where applicable | Good prognosis |
| Therapeutic Intervention | 9a | Types of therapeutic intervention (such as pharmacologic, surgical, preventive, self-care) | surgical |
| | 9b | Administration of therapeutic intervention (such as dosage, strength, duration) | Close follow-up for two years |
| | 9c | Changes in therapeutic intervention (with rationale) | NO |
| Follow-up and Outcomes | 10a | Clinician and patient-assessed outcomes (if available) | NO |
| | 10b | Important follow-up diagnostic and other test results | CT examination at 7 months showed no recurrence |
| | 10c | Intervention adherence and tolerability (How was this assessed?) | NO |
| | 10d | Adverse and unanticipated events | NO |
| Discussion | 11a | A scientific discussion of the strengths AND limitations associated with this case report | Complete imaging findings, clinical data and pathological findings are available; No genetic tests have been performed for th presence of mutations |
| | 11b | Discussion of the relevant medical literature **with references**. | YES |
| | 11c | The scientific rationale for any conclusions (including assessment of possible causes) | YES |
| | 11d | The primary "take-away" lessons of this case report (without references) in a one paragraph conclusion | YES |
| Patient Perspective | 12 | The patient should share their perspective in one to two paragraphs on the treatment(s) they received | Willing to undergo surgery |
| Informed Consent | 13 | Did the patient give informed consent? Please provide if requested | Yes ☑ No ☐ |

试用水印
